# Dimethyl fumarate improves white matter function following severe hypoperfusion: Involvement of microglia/macrophages and inflammatory mediators

**DOI:** 10.1177/0271678X17713105

**Published:** 2017-06-13

**Authors:** Jill H Fowler, Jamie McQueen, Philip R Holland, Yasmina Manso, Martina Marangoni, Fiona Scott, Emma Chisholm, Robert H Scannevin, Giles E Hardingham, Karen Horsburgh

**Affiliations:** 1 Centre for Neuroregeneration, University of Edinburgh, Edinburgh, UK; 2 Centre for Integrative Physiology, University of Edinburgh, Edinburgh, UK; 3 Current Address: Department of Basic and Clinical Neuroscience, Institute of Psychiatry, Psychology and Neuroscience, King's College London, London, UK; 4 Current Address: Developmental Neurobiology and Regeneration Lab, Parc Científic de Barcelona, Spain; 5 Current Address: Department of Health Sciences, University of Florence, Florence, Italy; 6 Biogen, Cambridge, Massachusetts, USA; 7 The UK Dementia Research Institute at The University of Edinburgh; 8 Centre for Cognitive Ageing and Cognitive Epidemiology, University of Edinburgh, Edinburgh, UK

**Keywords:** Electrophysiology, cerebrovascular disease, inflammation, microglia, white matter

## Abstract

The brain’s white matter is highly vulnerable to reductions in cerebral blood flow via mechanisms that may involve elevated microgliosis and pro-inflammatory pathways. In the present study, the effects of severe cerebral hypoperfusion were investigated on white matter function and inflammation. Male C57Bl/6J mice underwent bilateral common carotid artery stenosis and white matter function was assessed at seven days with electrophysiology in response to evoked compound action potentials (CAPs) in the corpus callosum. The peak latency of CAPs and axonal refractoriness was increased following hypoperfusion, indicating a marked functional impairment in white matter, which was paralleled by axonal and myelin pathology and increased density and numbers of microglia/macrophages. The functional impairment in peak latency was significantly correlated with increased microglia/macrophages. Dimethyl fumarate (DMF; 100 mg/kg), a drug with anti-inflammatory properties, was found to reduce peak latency but not axonal refractoriness. DMF had no effect on hypoperfusion-induced axonal and myelin pathology. The density of microglia/macrophages was significantly increased in vehicle-treated hypoperfused mice, whereas DMF-treated hypoperfused mice had similar levels to that of sham-treated mice. The study suggests that increased microglia/macrophages following cerebral hypoperfusion contributes to the functional impairment in white matter that may be amenable to modulation by DMF.

## Introduction

The integrity and connectivity of the brain’s white matter are critical in regulating efficient neuronal communication and maintaining cognitive function. The key functional cellular elements of white matter tracts are myelinated axons, the integrity of which is dependent on a constant supply of energy to enable accuracy and speed of action potential conduction between different brain regions.^1^ Myelinated axons and oligodendrocytes in white matter are highly vulnerable to reductions in cerebral blood flow.^2–4^ White matter hyperintensities, frequently detected in MRI scans from the brains of elderly individuals and those with dementia, are closely linked to cerebral hypoperfusion.^5,6^ A causal relationship has been shown in mouse models, whereby chronic hypoperfusion leads to white matter injury as detected using DT-MRI^7^ and pathological investigations which show impaired axon-glial integrity and robust microgliosis.^8,9^

At a functional level, the burden of white matter hyperintensity load has been correlated with cognitive deficits.^10,11^ We have demonstrated that chronic hypoperfusion in mice initially induces a selective deficit in spatial working memory^8^ that progresses to encompass deficits in spatial reference memory in the longer term.^12^ Oxygen-glucose deprivation in ex vivo brain slices induces an irreversible conduction failure of evoked compound action potential (CAP) assessed with electrophysiology in the corpus callosum, which is accompanied by axonal pathology and oligodendrocyte death.^13^

At a mechanistic level, an elevated inflammatory response is implicated in the pathogenesis of white matter pathology following cerebral hypoperfusion. We have reported increased numbers of microglia in white matter following mild chronic hypoperfusion^8,12,14^ which is also observed in more severe models.^15^ In white matter, increased levels of cytokines and chemokines such as TNFα, IL-1β, IL-6 and MCP1^16,17^ and increased levels of oxidised proteins and DNA are present following chronic hypoperfusion,^18–20^ which are both implicated in microglial-mediated damage.^9,21^ Pharmacological compounds that reduce activated microglia and pro-inflammatory pathways have beneficial effects on white matter after chronic hypoperfusion^22–24^ and confer protective effects against cognitive deficits.^25^ Similarly, drugs which reduce pro-inflammatory microglia in white matter can improve CAP amplitude after traumatic brain injury (TBI) and experimental autoimmune encephalitis (EAE; a mouse model of multiple sclerosis).^26,27^

Dimethyl fumarate (DMF), an anti-inflammatory drug,^28^ has neuroprotective effects in a number of mouse models of neurodegenerative diseases^29^ which are paralleled by a reduction in microgliosis.^30–32^ Similarly, DMF has neuroprotective effects in intracerebral or subarachnoid haemorrhage and focal cerebral ischaemia^33–36^ where it can improve neurological deficits and reduce microglial activation.^35,36^ DMF or monomethyl fumarate (MMF; the active metabolite of DMF) treatment of microglial cultures can inhibit lipopolysaccharide-induced production of pro-inflammatory cytokines and switch the molecular phenotype of microglia to an alternatively activated, neuroprotective one.^37–39^ Of relevance to white matter damage, DMF has been shown to reduce demyelination, axonal damage, and improve survival and disease course in EAE.^40–42^

The present study aimed to test the hypothesis that severe hypoperfusion would cause a functional and structural impairment of white matter and increased neuroinflammation and that DMF would improve white matter function following hypoperfusion by modulating neuroinflammation.

## Materials and methods

### Animals and surgery

Adult male C57Bl/6J mice (aged 3–4 months, group housed, obtained from Charles River UK, 12 h light/dark cycle, free access to food and water) underwent severe chronic cerebral hypoperfusion via bilateral common carotid artery stenosis using microcoils under isoflurane anaesthesia. A 0.18 mm internal diameter microcoil was applied to the left and a 0.16 mm microcoil was applied to the right common carotid artery as previously described.^15^ Sham mice underwent identical surgical procedure except the microcoils were not placed on the arteries. To assess if hypoperfusion could induce functional deficit in the corpus callosum and to determine the pathological correlates, CAPs were assessed in a cohort of mice using electrophysiology (hypoperfusion n = 12, sham n = 12). To determine the effects of DMF on white matter function and pathology, two experimental cohorts were studied. Cohort 1 underwent assessment of white matter pathology (sham vehicle n = 7; sham DMF n = 8; hypoperfused vehicle n = 15; hypoperfused DMF n = 11). Cohort 2 underwent electrophysiology and inflammatory-related multiplex analysis (sham vehicle n = 10; sham DMF n = 10; hypoperfused vehicle n = 16; hypoperfused DMF n = 13). Group size was determined using power calculations from electrophysiology data. Mice were closely monitored following surgery and those that had a poor recovery after surgery were culled. The initial and final cohort sizes are indicated in supplementary Table 1.

In all cohorts, mice were coded and randomly assigned to experimental groups. Mice were terminated seven days following surgery. The surgery was undertaken by an experimenter who was blinded to drug treatment group. The data analysis was conducted by researchers blinded to experimental grouping. All experiments were conducted under the UK Home Office Animals (Scientific Procedures) Act 1986, in agreement with local ethical and veterinary approval (Biomedical Research Resources, University of Edinburgh) and the ARRIVE guidelines.

### Laser speckle contrast imaging

Details of laser speckle contrast imaging are presented in the supplemental material.

### Administration of DMF

DMF (Sigma, UK) was administered twice daily (8 a.m. and 5 p.m.) by oral gavage at 100 mg/kg body weight in 0.8% hydroxypropyl methylcellulose (Sigma, UK) vehicle as previously described.^41^ Control animals received vehicle only. Administration of DMF or vehicle began 24 h prior to surgery and continued for seven days. Investigators administering DMF or vehicle were blinded as to surgical intervention.

### Electrophysiology

Mice were terminated by cervical dislocation followed by decapitation. Brains were rapidly dissected, placed in a cell strainer and submerged in ice-cold oxygenated artificial cerebrospinal fluid (aCSF) (189 mM sucrose; 10 mM D-glucose; 26 mM NaHCO3; 3 mM KCl; 5 mM MgCl2, 5; 0.1 mM CaCl2; 1.25 mM NaH2PO4) for 2–3 min. Brains were then affixed to a metal vibratome plate and placed in a bath of oxygenated ice-cold sucrose-aCSF. A single 400 µm coronal slice approximately −1.2–−1.7 mm from bregma was cut using a vibratome (Zeiss, Germany), and then transferred to a warmed incubation chamber (32–35℃) containing oxygenated aCSF (10 mM D-Glucose; 26 mM NaHCO3; 5 mM KCl; 2 mM CaCl2; 124 mM NaCl; 1.25 mM NaH2PO4; 1.3 mM MgSO4). The incubation chamber was then allowed to return to room temperature for at least 1 h prior to slice recording.

For recording, individual slices were transferred to the slice chamber, super-perfused with oxygenated room temperature aCSF (2–3 ml/min), secured with a slice anchor and allowed to rest for 30 min. Recording electrodes (1–5 MΩ) were pulled from borosilicate glass capillaries and filled with aCSF, connected to the head-stage via the Ag/AgCl wire and lowered into the corpus callosum. A bipolar stimulating electrode was connected to the stimulus isolation unit and lowered into the corpus callosum. Constant current square wave pulses were delivered at 0.2 Hz for eight sweeps and the average response of the final four sweeps used for analysis.^43^ The data were digitised at 200 kHz, amplified at 100× and low pass filtered at 5 kHz then recorded on Clampex 10.3.

CAPs were evoked, whilst holding the stimulus intensity constant (100 µs duration, 0.2 Hz), and recorded at 0.5 mm increments from 1 to 2.5 mm by moving the recording electrode. The post-stimulus latency of the CAP peak was then measured as a functional index of conduction velocity. For statistical comparison between groups, the CAP was measured at a distance of 2.5 mm apart.

Axonal health can be assessed by assaying changes in axonal excitability or axonal ‘refractoriness’.^43,44^ To assess the axon refractoriness, a single control stimulus was given followed by a paired pulse of equal intensity separated by a variable time window decreasing from 10 to 1.5 ms in 0.5 ms increments. The control amplitude was subtracted from the paired pulse response to leave only the second pulse which was measured and normalised to the % of the baseline CAP. The results were graphed versus the inter-pulse interval, and the interval that results in a 50% reduction in CAP was used for statistical comparisons.

### Tissue processing and immunohistochemistry

To determine the effects of severe hypoperfusion on white matter function and pathology, one cohort underwent electrophysiology, and an adjacent 2 mm slice of tissue was processed for paraffin embedding, then 8 µm sections were cut with a microtome. All other mice were deeply anesthetized with 5% isoflurane and transcardially perfused with 20 ml of 0.9% heparinized phosphate buffer saline (PBS) and then 20 ml of 4% paraformaldehyde (PFA) in 0.9% PBS seven days after surgery. After perfusion, brains were removed, postfixed in 4% PFA for 24 h and then transferred to 30% sucrose in PBS for 48 h. Brains were frozen in isopentane at −42℃ for 2 min. Thirty micrometres coronal sections were cut using a cryostat (Leica) and stored in cryoprotective medium (30% glycerol/30% ethylene glycol in phosphate buffer) at −20℃ until use. Cryostat sections were dehydrated through an increasing alcohol series, and then immersed in xylene for 10 min before being rehydrated through a decreasing alcohol series. Sections were then equilibrated in PBS for 5 min before antigen retrieval (if required). A 10 mM citric acid (pH 6) antigen retrieval was carried out for Iba1, MBP and MAG staining. Sections were blocked using 10% normal serum and 0.5% BSA for 1 h at room temperature. Primary antibodies were made up in blocking solution and sections were incubated in primary antibody at 4℃ overnight. The following day, the sections were incubated with secondary antibody in PBS for 2 h at room temperature. Coverslips were mounted using Vectashield HardSet mounting medium with or without DAPI. The following primary antibodies were used in the study: antimyelin-associated glycoprotein (MAG) (1:200, Abcam) as an index of axon-glial integrity; anti-myelin basic protein (MBP) to label myelin (1:100, Millipore); anti-ionised calcium-binding adaptor receptor molecule 1 (Iba1) to label microglia (1:100, Menarini); antiamyloid precursor protein (APP) to label axonal damage (1:200, Millipore); antiadenomatous polyposis coli protein CC1 (APC) to label oligodendrocyte cell bodies (1:200, Calbiochem); antineuronal-glial antigen (NG2) to label oligodendrocyte progenitor cells (1:100, Millipore). Alexa Fluor 488 and 555 conjugated secondary antibodies (1:500) were purchased from Life Technologies Ltd (UK).

### Image acquisition and analysis

All images were acquired using a confocal microscope (Leica TCS SP5) with either a 20× air or a 40× oil-immersion objective, pinhole of 1 Airy unit and 1024 × 1024 pixel resolution. MBP, MAG, APP, and Iba1 immunostainings were acquired as one single image, while CC1 and NG2 were acquired as a 5 µm stack. Images were acquired at the corpus callosum from the right hemisphere above the lateral ventricle at the stereotactic co-ordinates, lateral 2.40 ± 0.1 mm, Bregma −1.5 ± 0.1 mm. Quantitative measurements were performed using imageJ (FIJI) software. The number of Iba-1 positive cells was counted in three defined areas, and the average was used for statistical purposes. Mean fluorescence intensity (MBP, MAG immunostaining) or the percentage area of positive staining (Iba1 immunostaining) or number of cells/volume (CC1/NG2 immunostaining) were measured at the corpus callosum which was manually outlined using a free-hand tool. The number of swollen axons (axonal bulbs) in APP immunostained sections was counted in three defined areas along the corpus callosum and the average was used for statistical purposes. Observers were blind to surgery procedure and drug treatment.

### Inflammatory-related protein multiplex

Brain homogenates were assayed for inflammatory-related molecules using a Bio-plex mouse cytokine and chemokine assay (Biorad), which allows the detection of multiple inflammatory-related molecules in the same sample. A 1.8 mm section rostral to the brain slice used for electrophysiology was snap frozen in liquid nitrogen and stored at −80℃. Tissue was weighed and homogenised in 5 × volume of tissue lysing solution (Bioplex cell lysis kit, made according to manufacturer’s instructions), and then sonicated for 5 s at 10% amplitude. Samples were spun at 5000 *g* at 4℃, and then the supernatant was collected and stored at −80℃. Total protein concentration was determined using a Thermo Scientific Pierce BCA Protein Assay Kit and samples were diluted with bioplex cell lysis buffer to obtain a final total protein concentration of 13 mg/ml. Samples were then diluted to 8 mg/ml in bioplex sample buffer diluent before they underwent the multiplex assay according to the manufacturer’s instructions. The levels of seven inflammatory-related molecules were measured: interleukin-6 (IL-6), interleukin-1β (IL-1β), interferon-γ (IFN-γ), keratinocyte chemoattractant (KC), monocyte chemoattractant-1 (MCP-1), macrophage inflammatory protein 1-α (MIP-1α) and vascular endothelial growth factor (VEGF) in duplicate with standards (sham vehicle n = 8; sham DMF n = 8, hypoperfused vehicle n = 11; hypoperfused DMF n = 11). One animal from each of the hypoperfusion groups and two animals from each of the sham groups were randomly selected for omission from this experiment due to the space restrictions of the 96-well plate. Standards included in each kit were used to generate standard curves for each analyte, and then the mean fluorescence intensity for each analyte was calculated with five-point logistic regression using Bio-plex workstation software (Biorad).

### Statistics

Analysis and graphs were generated using Prism GraphPad 5.0 software. Data from the electrophysiology and related pathology cohorts were normally distributed and analysed using Student’s *t* test for independent samples. Associations between electrophysiology data and microglia numbers were analysed with Pearson’s correlation analysis. Other data, in which the effect of surgery and drug treatment or cerebral blood flow were investigated, were analysed by two-way analysis of variance (ANOVA) followed by Bonferroni post hoc test. Data were expressed as mean ± standard error of the mean and considered statistically significant when *p*-value was <0.05.

## Results

### Hypoperfusion induces severe reductions in cerebral blood flow

To determine if hypoperfusion induced reductions in cerebral blood flow, laser speckle contrast imaging was used one and seven days following surgery (supplemental Figure 1(a)). There was a significant effect of surgery (F_(1-18)_ = 58.2; *p* < 0.0001) and of time (F_(2, 36)_ = 51.6; *p < *0.0001) in the CBF values (% of baseline). Post hoc analysis showed that there is a significant reduction in CBF in hypoperfused mice compared with sham-treated mice 24 h (*p* < 0.001) and seven days following surgery (*p* < 0.001) (supplemental Figure 1(b)).

**Figure 1.**
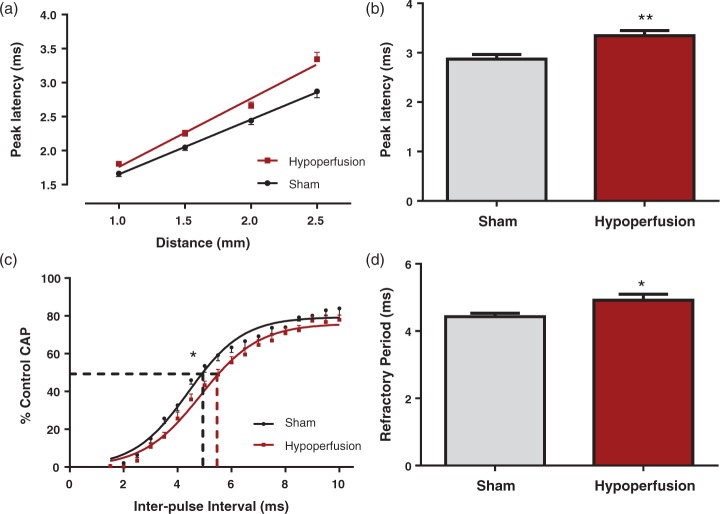
Deficits in white matter function in response to severe chronic hypoperfusion in myelinated fibres. (a, b) There was a significant increase in peak latency at 2.5 mm from the stimulating electrode (**p = 0.003), indicative of slowed conduction of myelinated fibres, in hypoperfused animals. (c, d) For axonal refractoriness, the interpulse interval resulting in a 50% reduction in the CAP was significantly reduced in hypoperfused mice, indicative of perturbed axonal health. (*p = 0.03), when compared with sham-treated animals. Data are presented as mean ± S.E.M. Student’s *t* test, *p < 0.05 **p < 0.01, n = 12 per group.

### Severe hypoperfusion induces a deficit in white matter function

At the outset, the effects of severe hypoperfusion on white matter function at seven days were assessed by electrophysiology in the corpus callosum. The peak latency of evoked CAPs, as an index of conduction velocity, was significantly increased in hypoperfused by 17% ± 3.6% as compared to sham mice (*p* = 0.003) (Figure 1(a) and (b)). As a measure of axonal health, axonal refractoriness of CAPs was studied in the corpus callosum. The interpulse interval resulting in a 50% reduction in the CAP was significantly increased in hypoperfused mice, indicative of perturbed axonal health (*p* = 0.03) when compared with sham-treated animals (Figure 1(c) and (d)). Collectively, these data indicate that callosal white matter fibres exhibit pronounced alterations in their electrophysiological properties in response to severe hypoperfusion.

### Increased white matter pathology and inflammation in response to severe hypoperfusion

**Figure 2.**
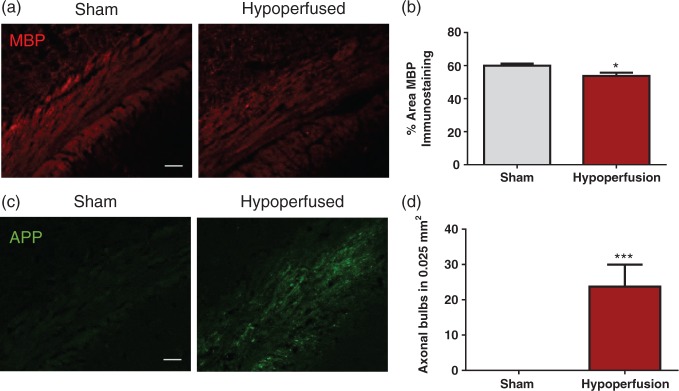
White matter pathology in response to severe chronic hypoperfusion. (a) Confocal images from the corpus callosum of animals immunostained with MBP (red), scale bar: 50 µm. (b) There was a significant reduction in the % area of MBP immunostaining following hypoperfusion (*p = 0.016). (c) Confocal images from the corpus callosum of animals immunostained with APP (green), scale bar: 50 µm. (d) There was a significant increase in axonal damage following hypoperfusion (***p = 0.001).

To determine if the deficit in white matter function was accompanied by pathological changes to myelinated axons and an inflammatory response, the adjacent slices to those used for electrophysiology were assessed. There was a significant reduction in the % area of MBP staining following hypoperfusion, indicating that hypoperfusion caused myelin pathology (*p* = 0.016; Figure 2(a) and (b)). Axonal bulbs, detected with APP immunohistochemistry, were not present in sham-treated animals but were significantly increased in hypoperfused animals (*p* = 0.001; Figure 2(c) and (d)), thus indicating that hypoperfusion caused axonal pathology.

**Figure 3.**
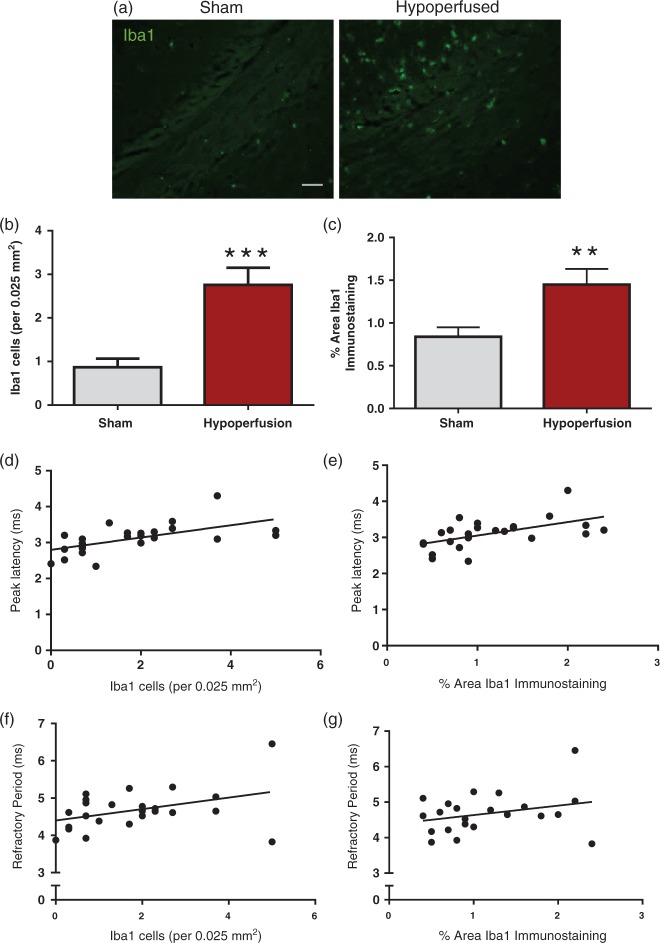
Elevated microglia/macrophage number and density in response to severe chronic hypoperfusion. (a) Confocal images from the corpus callosum of animals immunostained with Iba1 (green), scale bar: 50 µm. (b) There was a significant increase in microglial numbers following hypoperfusion (***p = 0.0003). (c) There was a significant increase in % area of Iba1 immunostaining following hypoperfusion (**p = 0.009). (d) There was a significant positive correlation between numbers of microglia and slowing of peak latency (r = 0.59, *p* = 0.002). (e) There was a signification positive correlation between % area of Iba1 immunostaining and peak latency (r = 0.55, *p* = 0.006). (f) There was no significant association between microglial numbers and slowing of axonal refractory period (r = 0.4, *p* = 0.06) (g) and no association between % area of Iba1 immunostaining and peak latency (r = 0.29, *p* = 0.17). Data are presented as mean ± S.E.M. Student’s *t* test, *p < 0.05 **p < 0.01, ***p < 0.001; n = 12 per group.

Iba-1-positive immunostaining was undertaken in the corpus callosum to investigate the impact of hypoperfusion on inflammation (Figure 3(a)). There was a significant increase in the number of Iba1-positive cells (*p* = 0.0003; Figure 3(b)) and the density of Iba1-immunostaining (*p* = 0.009; Figure 3(c)) following hypoperfusion. A number of studies have indicated that pharmacological drugs which reduce microglia in white matter can improve conduction velocity,^26,27^ suggesting that increased microglia may cause white matter dysfunction. We investigated this by correlating microglia/macrophages with peak latency or axonal refractoriness. There was a significant positive correlation between numbers of microglia and slowing of peak latency (r = 0.59, *p* = 0.002; Figure 3(d)) and density of Iba-1 immunostaining and slowing of peak latency (r = 0.55, *p* = 0.006; Figure 3(e)), thus indicating an association between microglia/macrophages and deficits in conduction velocity. There was no significant association between microglial/macrophage numbers and slowing of axonal refractory period (r = 0.4, *p* = 0.06; Figure 3(e)) or microglial/macrophage density and slowing of axonal refractory period (r = 0.29, *p* = 0.17; Figure 3(g)).

### DMF treatment improves white matter function following severe hypoperfusion

**Figure 4.**
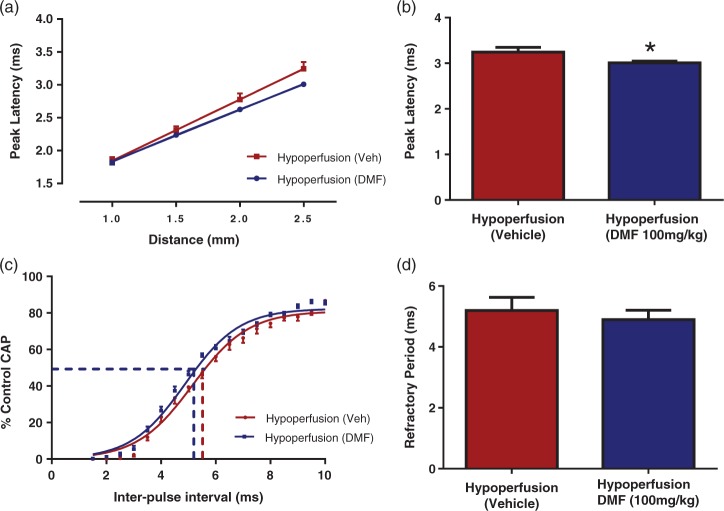
DMF treatment improved the peak latency of CAP, but not axonal refractoriness following severe chronic hypoperfusion. (a, b) DMF-treated hypoperfused mice had a reduced peak latency compared to vehicle-treated mice (*p < 0.05) indicating that DMF is able to improve conduction velocity of myelinated fibres. (c, d) Axonal refractoriness was not significantly altered by DMF treatment (*p* = 0.07). Data are presented as mean ± S.E.M. Student’s *t* test, *p < 0.05 n = 12 per group.

To further investigate the hypothesis that microglia can cause white matter dysfunction, a drug which is known to reduce microglial activation, DMF,^36^ was investigated to determine if it could protect improve white matter function following hypoperfusion. DMF significantly reduced the peak latency in hypoperfused mice by 7% ± 1.2% of vehicle-treated hypoperfused mice control values (*p* < 0.05) (Figure 4(a) and (b)). Thus, the data indicate a beneficial effect of DMF on conduction velocity of myelinated fibres. Axonal refractoriness was not significantly altered by DMF treatment (*p* = 0.07) (Figure 4(c) and (d)). Therefore, these data indicate that callosal white matter fibres exhibit pronounced alterations in conduction velocity and that DMF treatment can improve white matter function.

### Hypoperfusion causes a breakdown of axon-glial integrity that is unaffected by DMF

**Figure 5.**
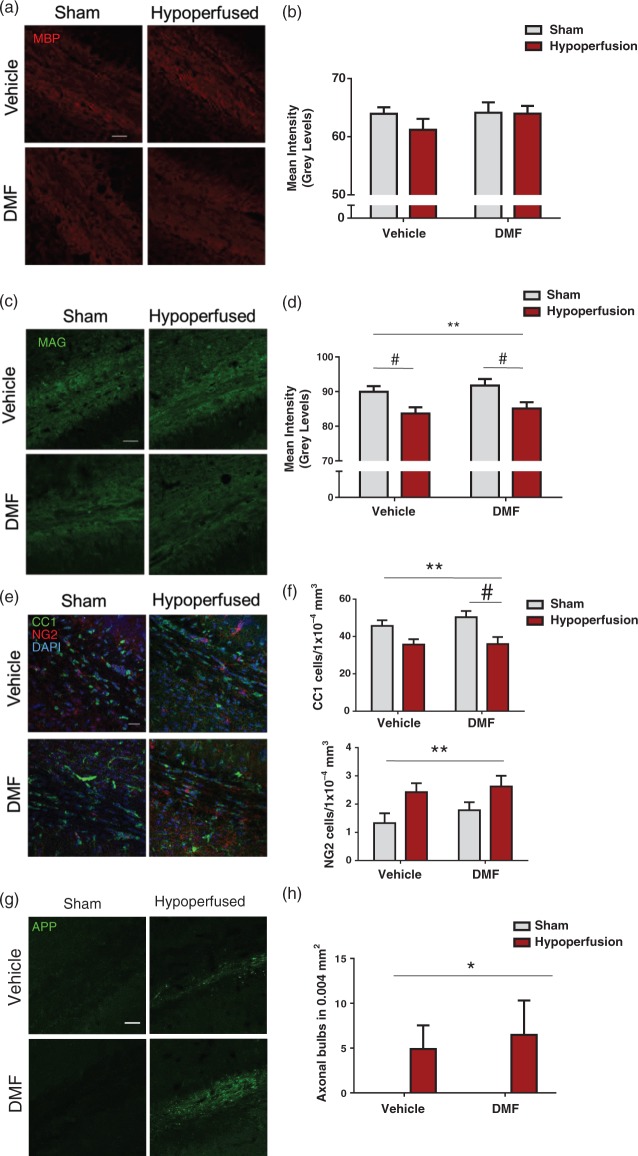
Axon-glial integrity was damaged by severe hypoperfusion but unaffected by DMF administration. (a) Confocal images from the corpus callosum of animals from four experimental groups immunostained with MBP (red), scale bar: 50 µm. (b) Quantification of MBP immunostaining sections showing no significant changes in myelin among the groups. (c) Confocal images from the corpus callosum of animals from four experimental groups immunostained with MAG (green), scale bar: 50 µm. (d) MAG immunostained sections showing changes in myelin in hypoperfused animals. Quantification of MAG mean intensity shows an overall significant effect of surgery (F_(1-31)_ = 11.7; **p = 0.002) Post hoc comparison shows a significant reduction with hypoperfusion in both vehicle (#*p* < 0.05) and DMF treated (#*p* < 0.05) groups. (e) Confocal images from the corpus callosum from four experimental groups immunostained for markers of mature oligodendrocytes (CC1) and oligodendrocyte precursor cells (NG2). Green: CC1; red: NG2, blue: DAPI, scale bar: 100 µm. (f) There is a significant reduction in the number of CC1 cells following hypoperfusion (F_(1-33)_ = 13.0; **p = 0.001) Post hoc comparison shows a significant reduction in the number of CC1 immunopositive cells following hypoperfusion in DMF-treated mice (#*p* < 0.05) but not in vehicle-treated mice (#*p* > 0.05). There was significant increase in NG2-positive cells following hypoperfusion surgery (F_(1-33)_ = 7.6; **p = 0.009). (g) APP-immunostained sections showing axonal damage in hypoperfused animals while minimal immunostaining is detected in shams. Green: APP. Scale bar: 50 µm. (h) Quantification of axonal bulbs shows an overall significant effect of surgery (F_(1-33)_ = 4.9; *p = 0.04). Data presented as mean ± SEM, Two-way ANOVA followed by Bonferroni post hoc test, sham vehicle n = 7; sham DMF n = 8; hypoperfusion vehicle n = 13; hypoperfusion DMF n = 9.

Following the demonstration that DMF improved white matter function in hypoperfused mice, its impact on hypoperfusion-induced pathology to myelinated axons was explored. MBP, a marker of myelin integrity, was not significantly altered in response to hypoperfusion or DMF administration (Figure 5(a) and (b)). Therefore, a further marker of myelin damage was investigated, MAG, as reductions in MAG intensity have been demonstrated previously in response to hypoperfusion, indicative of altered axon-glial integrity.^9,14^ A significant reduction in MAG immunostaining was determined following severe hypoperfusion (F_(1-33)_ = 11.7; *p* = 0.002), but there was no significant effect of DMF administration or interactions (Figure 4(c) and (d)). Post hoc comparison showed a significant reduction in MAG immunostaining with hypoperfusion in both vehicle- (*p* < 0.05) and DMF-treated (*p* < 0.05) groups when compared with sham controls.

Previous studies have revealed differential vulnerabilities of mature oligodendrocytes and their precursor cells in response to hypoperfusion. Oligodendrocytes have been suggested to facilitate axonal conduction by mechanisms other than myelination.^45^ Thus, the numbers of these cells were determined after hypoperfusion and DMF treatment using CC1 and NG2 immunohistochemistry for mature oligodendrocytes and OPCs, respectively (Figure 5(e)). Although there was a significant reduction in the number of CC1-positive cells (F_(1-31)_ = 13.0, *p* = 0.001) in response to hypoperfusion, DMF treatment had no effect and there were no significant interactions (Figure 4(f)). Post hoc comparison indicated a significant reduction in the number of CC1 immunopositive cells following hypoperfusion in DMF-treated mice (*p* < 0.05) but not in vehicle-treated mice (*p* > 0.05). In contrast, there was a significant increase in the number of NG2-positive cells (F _(1-33)_ = 7.6, *p* = 0.009); however, DMF treatment had no significant effect on the number of NG2 cells and there were no significant interactions (Figure 5(f)).

To build on the previous findings that axonal health was impaired by hypoperfusion but axonal refractoriness was not modulated by DMF, axonal pathology was investigated by APP immunostaining and assessment of the number of APP-positive axonal bulbs (Figure 5(g)). Axonal bulbs were not detected in sham mice but were observed in hypoperfused mice in the corpus callosum. There was a significant increase in the number of APP-positive axonal bulbs after hypoperfusion surgery (F_(1-33)_ = 4.9, *p* = 0.04), but the number was unaffected by DMF and there were no significant interactions (Figure 5(h)).

Thus, although hypoperfusion causes damage to myelinated axons, axon-glial integrity and the number of mature and immature oligodendrocytes, these measures were unaffected by DMF.

### Hypoperfusion increased microglial/macrophage density which is modulated by DMF

**Figure 6.**
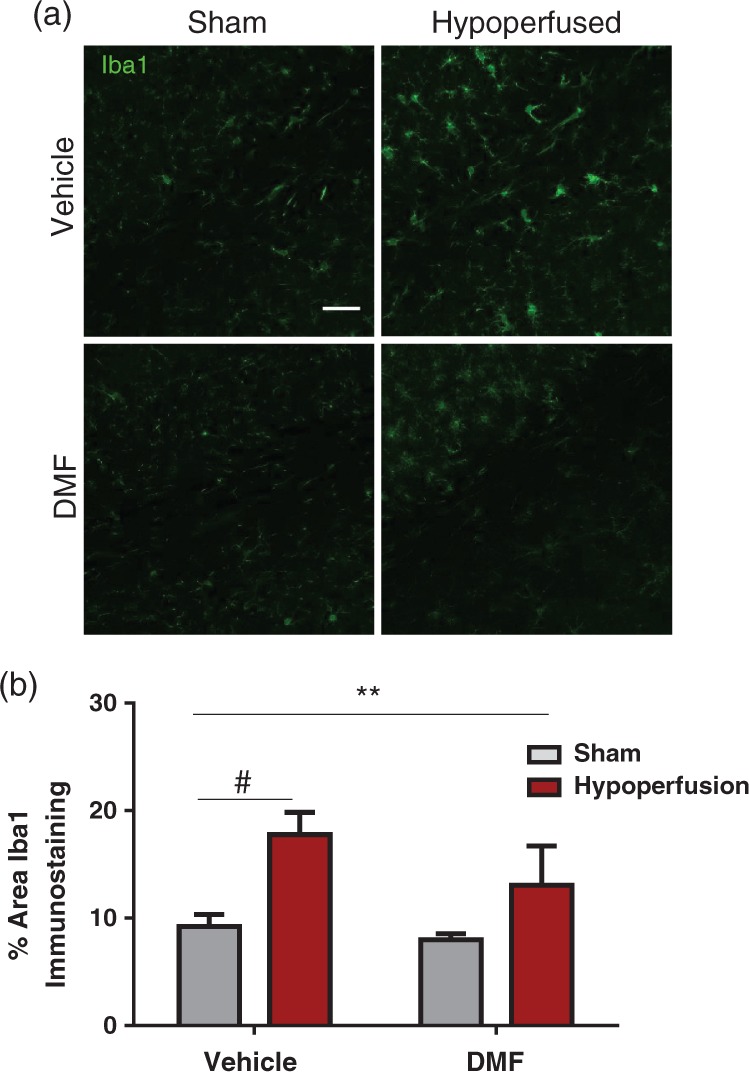
The effect of DMF on the microglial response. (a) Confocal images from the corpus callosum from four experimental groups. Green: Iba1; Scale bar: 50 µm. (b) There was a significant effect of surgery (F _(1-33)_ = 7.9; **p = 0.008) in the % area of Iba1 immunostaining. Notably, post hoc analysis showed that there is a significant increase in Iba1 staining in hypoperfused vehicle treated mice compared with sham vehicle treated mice (#*p* < 0.05), but no difference between sham and hypoperfused mice treated with DMF (*p* > 0.05). Data are presented as mean ± SEM, Two-way ANOVA followed by Bonferroni post hoc test, sham vehicle n = 7; sham DMF n = 8; hypoperfusion vehicle n = 13; hypoperfusion DMF n = 9.

Previous studies have indicated that DMF may exert protective effects through inflammatory mechanisms.^32,36^ Since we found a significant association between microglia/macrophages and deficits in white matter function, we next determined if microglial/macrophages would be affected by DMF treatment (Figure 6(a)). Microglial/macrophage density was significantly increased following hypoperfusion (F_(1-33)_ = 7.93, *p* = 0.008), but there was no effect of drug treatment (Figure 6(b)). Notably, post hoc analysis showed that there was a significant increase in Iba1 staining in hypoperfused vehicle-treated mice compared with sham vehicle-treated mice (*p* < 0.05), but there was no difference between sham and hypoperfused mice treated with DMF (*p* > 0.05), suggesting that DMF modestly reduced the density of microglia/macrophages.

### Hypoperfusion and DMF treatment effects on cytokines, chemokines and growth factors

The modest reduction in the microglial/macrophage response to hypoperfusion with DMF treatment suggested that DMF may act by modulating levels of inflammatory-related proteins. To further explore neuroinflammatory mechanisms, a range of cytokines, chemokines and growth factors were examined using a multiplex immunoassay.

**Figure 7.**
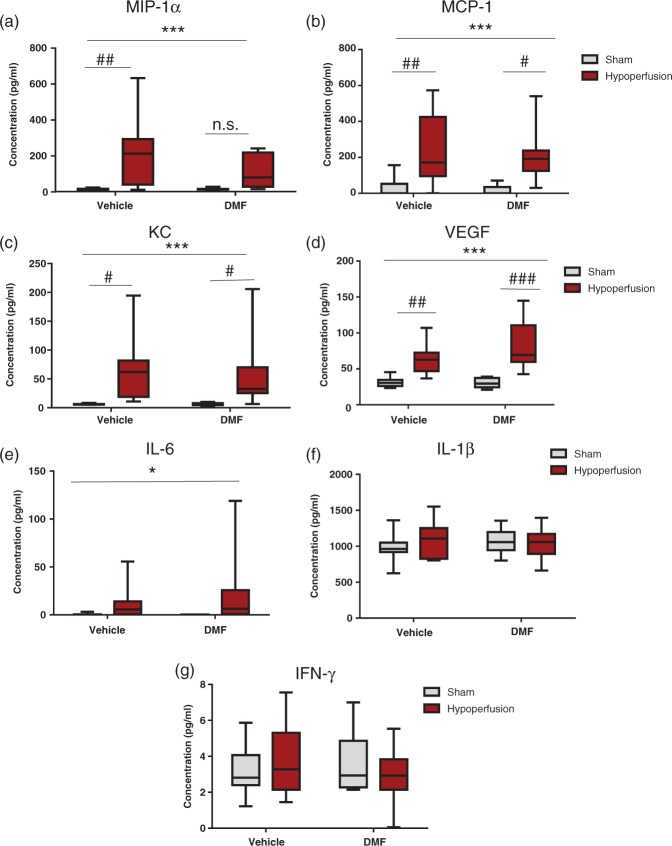
The effect of DMF on chemokines, growth factors and cytokines. Levels of inflammatory-related proteins were calculated (pg/ml) using a multiplex assay. (a) There was a highly significant effect of surgery (F _(1-34)_ = 13.7; ***p = 0.0008) in the concentration of MIP-1α (pg/ml). Post hoc analysis showed that there was a significant increase in MIP-1α levels in hypoperfused vehicle-treated animals compared with sham vehicle-treated animals (##*p* < 0.01); however, notably there was no significant increase in MIP-1a levels in the DMF-treated hypoperfused mice compared with the DMF-treated sham mice (*p* > 0.05). (b) There was a highly significant effect of surgery (F _(1-34)_ = 18.2; ***p = 0.0001) in the concentration of MCP-1 (pg/ml). Post hoc analysis showed that there was a significant increase in MCP-1 levels in hypoperfused vehicle-treated animals compared with sham vehicle-treated animals (##*p* < 0.01) and a significant increase in MCP-1 levels in hypoperfused DMF-treated animals compared with sham DMF-treated animals, but by a lesser magnitude (#*p* < 0.05). (c) There was a significant effect of surgery (F _(1-33)_ = 14.3; ***p = 0.0006) in the concentration of KC (pg/ml). Post hoc analysis showed that there was a significant elevation in KC levels following hypoperfusion vehicle compared with sham vehicle treatment (#*p* < 0.05), and in hypoperfused DMF-treated mice when compared with sham DMF-treated mice (#*p* < 0.05). (d) There was a highly significant effect of surgery (F _(1-34)_ = 34.8; ***p < 0.0001) in the concentration of VEGF (pg/ml). Post hoc analysis showed that there was a significant increase in VEGF levels in hypoperfused vehicle-treated animals compared with sham vehicle-treated animals (##*p* < 0.01). Notably, there was also a significant increase in VEGF levels in the DMF-treated hypoperfused mice compared with the DMF-treated sham mice, by a greater magnitude (###*p* < 0.001) than the increase seen in vehicle treated mice. (e) For IL-6 levels, there was a significant effect of surgery (F _(1-34)_ = 4.4, *p = 0.04). (f and g) There was no significant effect of hypoperfusion or DMF for IL-1β (f) or for IFN-γ (g). Data are presented as mean ± SEM, two-way ANOVA followed by Bonferroni post hoc test, sham vehicle n = 8; sham DMF n = 8; hypoperfusion vehicle n = 11; hypoperfusion DMF n = 11.

The levels of chemoattractant and growth factor molecules MIP-1α, MCP-1, KC and VEGF were examined firstly (Figure 7). All four molecules were significantly increased after hypoperfusion (MIP-1α F_(1-34)_ = 13.7, *p* = 0.0008; MCP-1 F_(1-34)_ = 18.2, *p* = 0.0001; KC F_(1-33)_ = 14.3, *p* = 0.0006; VEGF F_(1-34)_ = 34.8, *p* < 0.0001), and there was no significant effect of DMF administration or significant interactions. Post hoc comparison showed a significant increase in the levels of MCP-1, KC and VEGF in hypoperfused vehicle-treated mice compared with sham vehicle-treated mice and in DMF hypoperfused compared to DMF sham-treated mice. In contrast, although MIP-1α levels were significantly increased (*p* < 0.01) in hypoperfused vehicle-treated mice compared with sham vehicle-treated mice, MIP-1α levels in DMF-treated hypoperfused mice were not significantly different compared with sham DMF-treated mice (*p* > 0.05) suggesting that DMF reduced levels of MIP-1 α (Figure 7(a)).

Three cytokines were examined, IL-6, IL-1β and IFN-γ (Figure 7). IL-6 levels were significantly increased after hypoperfusion (F_(1-34)_ = 4.4, *p* = 0.04) but unaffected by DMF treatment, and there were no significant interactions (Figure 7(e)). IL-1 β and IFN-γ were unaffected by hypoperfusion surgery or DMF treatment (Figure 7(f) and (g)).

Therefore, overall analyses indicated hypoperfusion had a prominent effect on inflammatory-related molecules which was unaffected by DMF treatment except for MIP 1α levels which were dampened by DMF treatment.

## Discussion

The present study demonstrated that severe hypoperfusion induces a deficit in white matter function in the corpus callosum which is correlated with a marked increase in microglia/macrophages. Treatment with an anti-inflammatory drug, DMF, improved white matter function and dampened microglial/macrophage density induced by severe hypoperfusion.

White matter function assessed with electrophysiology was markedly impaired in the corpus callosum, key tracts connecting cerebral hemispheres and vulnerable to damage following chronic hypoperfusion.^8,15^ The assessment of evoked CAPs has been used in a number of other studies to demonstrate that perturbations in white matter can impact their function. For example, deficits in white matter function have been detected by measuring CAP in response to oxygen glucose deprivation,^46–48^ TBI^49^ and excitotoxicity.^50^ This approach is very sensitive to the electrophysiological properties of white matter and can be used to detect altered function caused by subtle alterations of white matter such as differing effect of sex hormone on recovery from cuprizone demyelination^51,52^ and glutathione depletion.^53^

The deficits in white matter function induced by hypoperfusion included a slowing of peak latency of CAP, indicative of slowed conduction velocity. Conduction velocity is the rate at which a nerve impulse is propagated, and is dependent on normal myelination. Similar slowing of conduction velocity has been demonstrated following low-level blast trauma,^54^ demyelination^44^ and neonatal hyperoxia.^55^ We also demonstrated that severe hypoperfusion increased the axonal refractory period, indicative of perturbed axonal health. Increased refractory period has been reported after hypoxia in spinal cord; however, this could be recovered following re-oxygenation, suggesting that hypoxia may be a key mechanism of increased refractoriness following hypoperfusion.^56^ Demyelination of axons induced by cuprizone or TBI and compression injury also cause increases in refractoriness.^44,49,57,58^

Severe hypoperfusion resulted in white matter pathology, including myelin and axonal damage detected with MBP and APP immunohistochemistry that may account for the deficits in white matter function. This is an agreement with other studies where structural alterations in white matter are reported to underlie functional alterations. For example, demyelination or myelin thinning and axonal damage, detected with electron microscopy or APP immunohistochemistry, are present in white matter following cuprizone demyelination, TBI or excitotoxicity which resulted in reduced CAP amplitude, conduction velocity and increased refractoriness.^43,44,49,50,57^

We found a significant elevation in microglia/macrophages in the corpus callosum following severe hypoperfusion. Microglial activation is a robust response to reduced cerebral blood flow following chronic hypoperfusion^8,14^ and in stroke models.^59,60^ Furthermore, there was a positive correlation between microglia/macrophages and slowing of peak latency, suggesting that the elevated inflammatory environment in myelinated axon tracts may contribute to the functional deficit. Increased microglial levels are also reported in white matter that exhibit deficits in conduction velocity or refractoriness following cuprizone demyelination,^44^ EAE^61^ or TBI.^58^ The extent of axonal damage in demyelinating MS lesions is associated with increased numbers of activated microglia/macrophages^62^ which have been hypothesised to play a mechanistic role in white matter damage through the release of inflammatory-related mediators such as nitric oxide, which can induce reversible conduction block and axonal degeneration in demyelinated axons.^63^

To further test the hypothesis that microglia may mediate the deficits in white matter function, we used a drug, DMF, which is known to reduce microgliosis in a number of disease models including focal cerebral ischaemia.^36,64^ DMF was found to significantly improve peak latency following severe hypoperfusion. This corroborates with other studies which have shown that DMF can exert protective effects on motor and neurological function in focal cerebral ischaemia,^36,64^ intracerebral haemorrhage^35^ and other models of neurodegeneration.^29,31,32,41,65^ DMF is also known to protect against cognitive deficits assessed in the Morris water maze in a mouse model of subarachnoid haemorrhage.^34^ MMF (derivative of DMF) protects against functional deficits assessed with electrophysiology in retinal ischaemia-reperfusion^66^ and in an experimental autoimmune neuritis model.^65^

Although DMF exerted protective effects on white matter function in the corpus callosum, this was not paralleled by protective effects on white matter structure. In a first cohort, reduced MBP immunostaining was determined in mice that exhibited functional deficits following hypoperfusion, confirming the findings of Miki et al.^15^ in severe hypoperfusion. However, in a second cohort, a similar reduction in MBP staining was not found. The reasons for these differences between cohorts are not clear. One explanation may be that the tissue was processed differently using sucrose treatment and cryostat sectioning as opposed to paraffin embedding. To further examine myelin integrity after hypoperfusion, we therefore used another marker, MAG which we previously demonstrated to be sensitive to chronic hypoperfusion.^9,14^ However, despite the reductions in MAG immunostaining in response to severe hypoperfusion, MAG pathology was not attenuated with DMF. Oligodendrocytes contain a number of ion channels and neurotransmitter receptors which monitor neuronal activity and facilitate axonal conduction by mechanisms other than myelination.^45^ Therefore, in light of the fact that DMF did not modulate MAG pathology, we examined the effects of DMF on mature and immature oligodendrocyte populations. We demonstrated that mature oligodendrocytes are particularly vulnerable to hypoperfusion with levels markedly reduced seven days following hypoperfusion. This finding is in agreement with our previous study^14^ and that of others^18^ indicating CC1 levels are reduced within days following chronic hypoperfusion and focal cerebral ischemia.^67,68^ In contrast, the levels of NG2 oligodendrocyte cells were increased in response to severe hypoperfusion, as others^69^ have reported following focal cerebral ischaemia. However, there was no protective effect of DMF administration on oligodendrocyte numbers after hypoperfusion. Similarly, DMF has no protective effects on mature and oligodendrocyte precursor cells following cuprizone demyelination.^70^

Collectively, the data indicate that DMF can improve white matter function following hypoperfusion, but this is not associated with a concomitant protective effect on axonal-glial integrity. A multitude of factors contribute to conduction velocity along myelinated fibres which also include the distribution and density of ion channels at nodal regions. Other studies have showed that deficits in conduction velocity and axonal refractoriness are sustained in the recovery from neonatal hyperoxia or withdrawal from cuprizone diet,^44,55^ despite a recovery of axon-glial integrity. Instead, they found sustained, subtle alterations in the structural composition of the paranode and Node of Ranvier including increased spread of Nav1.6 channels along the Node of Ranvier, which we have also reported after mild chronic hypoperfusion.^9^ Clofazimine, a drug which prevents loss of potassium channels, can improve conduction velocity and axonal refractoriness following TBI.^58^ Therefore, the limited exploration of axon-glial integrity that we have carried out may not be the most sensitive pathological indices of functional deficits, and instead specific regions of node and paranodal domains of myelinated axons and ion channels may be vulnerable.

An important consideration of the present study is whether the dose of DMF was appropriate to ensure maximal protective effects in white matter. Protective effects of DMF have been reported at doses as low as 15 mg/kg in intracerebral/subarachnoid haemorrhage and EAE^33,34,40^ and 30 mg/kg in a model of Huntington’s disease.^29^ More recently, Schulze-Topoff et al.^42^ used the same dose as the present study (100 mg/kg) to demonstrate clinical and histological protection following EAE. DMF is thought to act mechanistically by enhancing the activity of Nrf2 signalling, an endogenous survival pathway with anti-oxidant and anti-inflammatory effects. Nrf2 is a transcription factor that activates a battery of cytoprotective genes such as genes encoding heme-oxygenase-1 (*hmox1*), osgin 1 (*osgin 1*) and NAD(P)H dehydrogenase quinone 1 (*nqo1*). Brennan et al.^71^ recently carried out an investigation of the dose–response effect of DMF on Nrf2-dependent gene changes. For many genes studied, such as *hmox1*, there were no notable increases in the brain following 100 mg/kg of DMF; however, 400 and 600 mg/kg could induce increases in *hmox1* expression, an effect reported by others.^32^ Therefore, it may be possible that the modest protective effects that DMF (100 mg/kg) had on white matter function in the present study could be enhanced with a higher dosage.

In contrast to the protective effects of DMF on peak latency, we found no parallel protective effects on axonal refractoriness, as an index of axonal health. Similarly, we found a significant increase in APP immunostaining, indicative of axonal damage, following severe chronic hypoperfusion that was not attenuated with DMF treatment. Similar results were reported on white matter function following administration of the immunophilin ligand FK506 after TBI. FK506 treatment could protect against deficits in CAP amplitude, but did not reduce refractory changes in myelinated fibres.^72^ DMF has been reported to protect against axonal damage detected with APP immunohistochemistry in EAE^41^ and EAN;^73^ however, this may reflect a different disease mechanism.

Although we did not detect protective effects of DMF on hypoperfused-induced damage to myelinated axons, we found that DMF led to modest reductions in the extent of microglial/macrophage density. There is a marked reduction in microglial levels in vivo following DMF treatment in a mouse model of synucleinopathy^32^ and Parkinson’s disease.^31^ Of relevance to the current study, DMF was found to significantly reduce microglial levels following focal cerebral ischaemia,^36^ an effect that was paralleled by reduced cytokine levels.^64^ In support of our hypothesis that reductions in microglia/macrophages can improve white matter function, HDAC inhibition or the sodium channel blocker safinamide reduced pro-inflammatory microglia/macrophages and improved CAP amplitude in the corpus callous following TBI or EAE.^26,27^ One limitation of the current study is that the Iba-1 immunostaining cannot discriminate between macrophages and brain-derived microglia; therefore, some of the immunostaining may represent infiltration of peripheral-derived macrophages. Indeed, transient blood–brain barrier disruption has been reported in white matter in a rat model of chronic cerebral hypoperfusion^74^ and may contribute to the functional deficit in the present study. In support of this, it has been reported that DMF can protect the blood–brain barrier following focal cerebral ischaemia in the mouse^75^ via decreased matrix metalloproteinase activity and protection of interendothelial tight junctions, resulting in reduced brain oedema. This raises the possibility that the beneficial effects of DMF on white matter function that we have reported may have been partly mediated through protection of the blood–brain barrier, particularly given the modest effects of DMF on inflammation that we found.

DMF has been shown to inhibit microglial inflammation in vitro and to suppress the expression of pro-inflammatory cytokines.^76^ Therefore, to further investigate the modulation of inflammatory-related proteins after severe hypoperfusion and DMF treatment, a panel of molecules were investigated with multiplex. The chemoattractant molecules MIP-1α (CCL3) and MCP-1 (CCL2) are both upregulated in the present study, and are also upregulated in response to focal cerebral ischaemia and chronic hypoperfusion.^77–79^ These chemokines regulate the infiltration of monocytes/macrophages into the CNS under pathological conditions and are associated with exacerbating neuronal damage,^80^ whereas their inhibition protects neurons.^81^ We have reported that MIP-1α levels are modestly dampened following DMF administration, and this may contribute to the protective effect of DMF on white matter function, possibly via effects on monocyte/macrophage infiltration from the periphery. Lin et al.^64^ reported that DMF reduced the number of MPO + neutrophils and CD3 + T cells which infiltrated the penumbral region following focal cerebral ischaemia.

Levels of the growth factor VEGF were increased in response to severe chronic hypoperfusion. VEGF is associated with protective effects following focal cerebral ischaemia and chronic cerebral hypoperfusion, including neuroprotection, increased angiogenesis and cerebral blood flow.^82,83^ Of interest, Wiesner et al.^84^ showed that VEGF levels are elevated in astrocyte cultures treated with DMF, mediated by Nrf2 signalling.

Of the three cytokines that were assessed in the current study, only IL-6 was found to be significantly increased seven days following chronic hypoperfusion. IL-6 is reported to have both pro- and anti-inflammatory effects;^85^ however, in the context of cerebral hypoperfusion, it confers neuroprotective effects.^86^ However, we did not find evidence that DMF altered IL-6 levels. In contrast to the increase in IL-6 we have reported, the cytokines IL-1β and IFN-γ were not altered in response to chronic hypoperfusion or DMF treatment. IL-1β is increased after focal cerebral ischaemia^87^ and promotes neuronal damage. Although we did not detect any alterations in IL-1β, others have reported that levels of IL-1β appear to alter transiently at different time points in response to differing extents of cerebral hypoperfusion.^16,88^ We also reported no alterations in IFN-γ levels, which concurs with a findings in focal cerebral ischaemia.^89^ Collectively, there was minimal evidence that DMF could modulate cytokine levels, whereas modest effects were seen on MIP-1α.

Given the modest protective effects of DMF that we have reported seven days following severe hypoperfusion, it would be interesting to study longer term time points. Miki et al.^15^ showed that severe hypoperfusion causes progressive white matter pathology and we have previously reported that more modest decreases in cerebral blood flow cause increased microgliosis and altered axon-glial integrity at one month,^8,9^ that progresses to include microinfarcts, haemorrhage and blood–brain barrier alterations. DMF is reported to reduce ischaemic neuronal death, oedema and blood–brain barrier breakdown^36,64,75^ in the acute response to focal cerebral ischaemia thus may have protective effects in the chronic response to modest blood flow reduction. DMF is reported to have protective effects following long-term administration in EAE,^40,41^ Parkinson’s disease,^32^ and following one year of administration in a mouse model of Huntington’s disease.^29^

To conclude, we have demonstrated that severe hypoperfusion caused deficits in white matter function and that DMF improved white matter function following severe hypoperfusion. Although there was no effect of DMF on the structural integrity of damaged myelinated axons, DMF had modest effects on microglia/macrophages.

## Supplementary Material

Supplementary material
