# Supplementary material for: Dimethyl fumarate improves white matter function following severe hypoperfusion: Involvement of microglia/macrophages and inflammatory mediators
Source: J Cereb Blood Flow Metab. 2017 Jun 13;38(8):1354–70. doi: 10.1177/0271678X17713105 (PMC6077928; doi:10.1177/0271678X17713105)
Supplement: Supplementary material [file JCB713105_supplementary_material.pdf]

SUPPLEMENTARY DATA

Table S1:

	Sham		Hypoperfusion	
	Vehicle	DMF	Vehicle	DMF
Cohort 1	**7**	**8**	**13** (2)	**9** (2)
Cohort 2	**10**	**10**	**12** (4)	**12** (1)

SUPPLEMENTAL TABLE LEGEND:

Table S1: The survival rate of vehicle and DMF-treated animals

Final cohort sizes for cohort 1 (pathology) and cohort 2 (electrophysiology and inflammatory-related protein multiplex). The numbers of animals that were culled due to poor response are indicated in parenthesis. The survival rate of vehicle and DMF-treated animals was examined to determine whether DMF improved survival following severe hypoperfusion. Survival rate in vehicle-treated hypoperfused mice was 80.6%, in line with previously reported findings (Miki et al.,2009) [15]. The survival rate of DMF-treated animals was approximately 87.5% however this was not significantly different (p= 0.458, log-rank test).

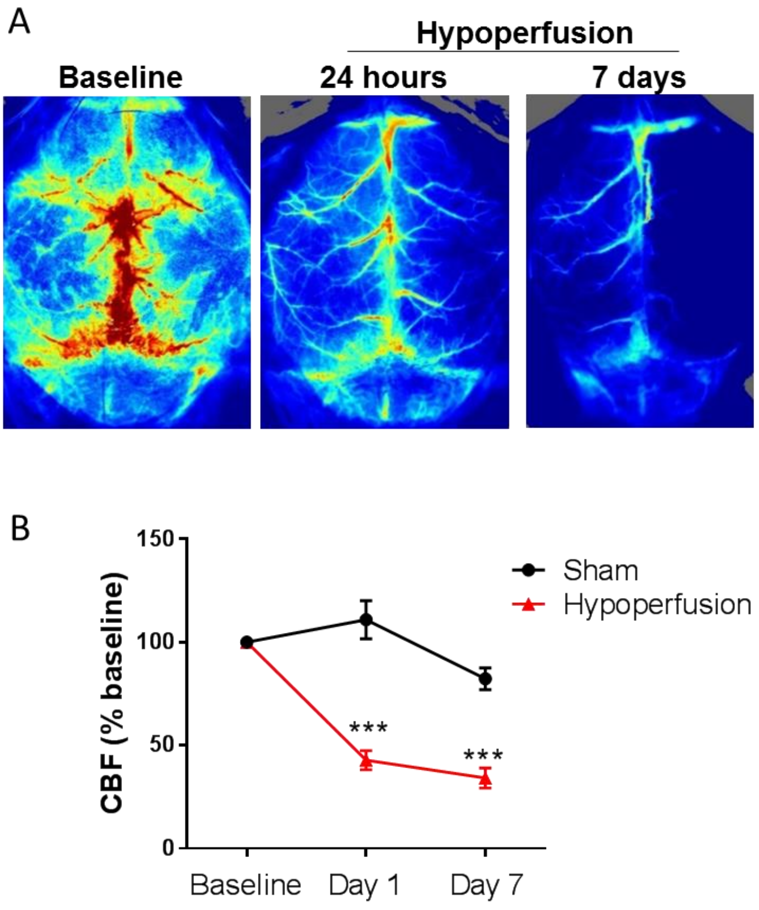

Figure S1: Cerebral blood flow assessed with laser speckle flowmetry was reduced following severe hypoperfusion

(A) Representative laser speckle images showing cortical blood flow imaged through the intact skull of mice at baseline and following chronic hypoperfusion. (B) Quantification of cerebral blood flow (expressed as a % of baseline value per animal). There was a significant effect of surgery ($F_{(1-18)}$= 58.2, p< 0.0001,) and of time ($F_{(2, 36)}$= 51.6, p< 0.0001,) in the CBF values (% of baseline). *Post-hoc* analysis showed that there is a significant reduction in CBF in hypoperfused mice compared with sham treated mice 24 hours after surgery (***p< 0.001) and 7 days following surgery (***p< 0.001).

SUPPLELEMENTAL METHODS SECTION

Cortical blood flow was measured in the right hemisphere by laser speckle imaging 24 hour prior to surgery and at day 1 and 7 after surgery. Animals were anaesthetised with 5% isoflurane in oxygen in an anaesthetic chamber before being transferred to a stereotaxic frame where the head was fixed into position. Anaesthesia was maintained at 2-2.5% isoflurane via a nose cone and body temperature constantly monitored using a rectal probe and maintained at 37°C using a heating blanket. An incision was made down the midline of the head to expose the skull ultrasound gel was applied to prevent drying. A moorFLPI2 Speckle Contrast Imager (Moor Instruments, UK) connected to a laptop computer was positioned 20 cm from the brain. Images were acquired using moorFLIP2 Measurement software (v1) at a resolution of 752 x 580 pixels and a frequency of 25 frames/second. Stable baseline blood flow in the barrel cortex was recorded for 2 minutes. Speckle contrast images were analysed using MoorFLPI-2 Review software (version 4.0).
